# A usability and feasibility study of a computerized version of the Bath Adolescent Pain Questionnaire: the BAPQ-C

**DOI:** 10.1186/s12887-019-1899-3

**Published:** 2020-01-06

**Authors:** Abbie Jordan, Fiona M. Begen, Lisa Austin, Rhiannon T. Edwards, Hannah Connell

**Affiliations:** 1 Department of Psychology & Centre for Pain Research, University of Bath, Bath, BA2 7AY UK; 2 Department of Psychology, University of Bath, Bath, BA2 7AY UK; 3 Department of Health, University of Bath, Bath, BA2 7AY UK; 4 Faculty of Arts and Social Sciences, The Open University, Walton Hall, Milton Keynes, MK7 6AA UK; 5 Bath Centre for Pain Services, Royal United Hospitals Foundation Trust, Bath, BA1 1RL UK

**Keywords:** Assessment, Adolescent chronic pain, Electronic, Questionnaire, Measures, Impact: computerized, Acceptability, Feasibility, Usability

## Abstract

**Background:**

Pain is a common experience in adolescence, with up to 44% of adolescents reporting chronic pain. For a significant minority, severe pain becomes an ongoing disabling problem. Treatment of adolescent chronic pain aims to reduce the impact of pain on adolescents’ lives. Efficient, accurate assessment of the impact of pain is essential to treatment. The ‘Bath Adolescent Pain Questionnaire’ (BAPQ) is a psychometrically robust multidimensional self-report measure of adolescent functioning. Whilst widely used, the paper-based format of the BAPQ can present completion difficulties for adolescents experiencing chronic pain. To increase the accessibility and clinical utility of the BAPQ, an electronic version of the measure is needed. This study assesses the usability and feasibility of a computerized version of this measure (BAPQ-C) in an adolescent chronic pain population.

**Methods:**

Fourteen adolescents (13 females; 13–16 years) were recruited from a hospital-based residential pain management programme. Participants completed a qualitative ‘thinking aloud task’ whilst completing the BAPQ-C. and, an acceptability questionnaire regarding the BAPQ-C. Data were analysed using thematic analysis, a widely used qualitative method of data analysis .

**Results:**

Two themes labelled ‘engagement and technological appeal’ and ‘accessibility and independence’ were generated. Themes revealed numerous factors contributing to participants’ preference for the BAPQ-C compared with the paper version of the BAPQ. Participants reported that the BAPQ-C was ‘quicker’ and ‘easier’ to complete than the BAPQ. Functional aspects of the BAPQ-C which included use of a touch screen rather than a pen and paper, font colours/styles, the zoom function and the spellchecker, provided participants with improved access. This subsequently increased participants’ independence and confidence when completing the measure.

**Conclusion:**

The BAPQ-C is a feasible multidimensional tool for the assessment of functioning in adolescents who experience chronic pain. It was well-received by participants who were able to complete the measure more quickly, independently and confidently than the paper-based BAPQ. Increased speed, ease and accuracy of completion make the BAPQ-C an ideal tool for use in busy clinical and research settings. Findings highlight the potential benefits of adopting the BAPQ-C when assessing the impact of chronic pain on adolescents in clinic and home-based settings.

## Background

Pain is a common experience in adolescence [1–4] . Although most pain in this particular age group is clinically unremarkable, for a significant minority, pain becomes a chronic and disabling problem [3]. For these adolescents, the impact of ongoing pain is wide-ranging and all encompassing. Adolescents who experience chronic pain report impaired functioning across numerous domains including physical, psychological, social and developmental functioning [5–7].

Treatment of adolescent chronic pain aims to reduce disability and the impact of pain on these varying domains of adolescents’ lives [8]. A critical element in the treatment of adolescent chronic pain concerns assessment of the impact of pain, with adolescent self-reports of their pain experiences providing greater understanding of the day-to-day functional and social impairments associated with chronic pain [9, 10]. Additionally, adolescent self-report plays a significant role in informing initial diagnoses and ongoing treatment evaluation, and is crucial in the assessment of interventions to manage pain and associated disability [11–13]. Whilst the measurement of the impact of adolescent chronic pain is an important clinical task [11], there is little consensus regarding the use of appropriate measurement tools with this distinct population. A comprehensive review of adolescent measures in the context of chronic pain identified the need for psychometrically robust multidimensional assessment tools to assess adolescent and parental functioning [14]. The Bath Adolescent Pain Questionnaire (BAPQ) [15] was developed in response to these particular needs. The BAPQ is a 61 item multidimensional self report measure which assesses adolescent functioning in a range of domains including physical, psychological, family, social and developmental functioning. The measure has proven to be successful, psychometrically robust and clinically useful, with adoption in a wide range of UK and international settings [15–18].

Despite the popularity of the BAPQ, adolescents and clinicians have called for a computerized version of the paper based measure. Such demands fit with developments suggesting an increase in the use of technology to complete health related self-report assessment measures; particularly in adolescent populations [19, 20]. Electronic assessment tools such as personal digitial assistants, electronic pain diaries, smartphone and web based pain assessments have been successfully developed for use by adolescents [19, 21–23]. Alongside these novel electronic assessments of adolescent chronic pain, it is important to consider the adaptation of existing psychometrically robust paper measures into electronic measures. In wider populations, the use of health related measures converted from paper to electronic formats has shown distinct advantages. Typically, studies have addressed issues of both usability (intuitiveness of the user interface) and feasibility (compliance and acceptability) of such electronic versions of paper based measures [21]. Study findings have shown that high levels of patient acceptability and compliance, low adminstrative burden, and avoidance of secondary data entry errors have been reported [24–26]. A primary concern when adapting existing paper measures to electronic format is retention of the psychometric properties of the original measure, and whether electronic versions can be deemed directly comparible to paper versions. Extensive reviews regarding the ‘equivalence’ of paper and electronic measures [26–28] suggest that electronic measures can indeed be considered equivalent to their paper based counterparts where only minor alterations to a measure have been made in order to accommodate electronic formats [24, 27].

There is a clear need to develop psychometrically robust electronic pain assessment measures that are both acceptable to adolescents completing the measures and useful for clinicians undertaking assessment [11, 19]. Whilst the BAPQ has shown great promise for use in a research capacity, its use in clinical settings and acceptability to adolescents is limited when administered as a paper based measure. In direct response to these issues and given the extensive use and popularity of electronic media amongst adolescents [29], a computerized version of the BAPQ (known as the BAPQ-C) was developed for real time completion by adolescents in clinical settings.

### Aims and objectives

The overall aim of the study was to establish the usability and feasibility of a computerized version of the BAPQ (BAPQ-C) in a population of adolescents experiencing chronic pain.

The immediate objectives of the study included:

1. Administration of the BAPQ-C to fourteen adolescents with chronic pain in a residental treatment clinical setting.
2. Evaluation of participants’ responses to the electronic format of the BAPQ-C through participation in cognitive debriefing.
3. Examination of the usability of the BAPQ-C for adolescents via completion of a usability questionnaire concerning BAPQ-C completion.

## Methods

### Design

A predominantly qualitative design was adopted to examine adolescents’ experiences and perceptions of use of the BAPQ-C. A limited amount of quantitative data was collected via the Likert scales to enable participants to report their perceptions of the acceptability of the BAPQ-C. Following recommendations of Coons et al. [24] concerning computerized adaptation of paper based measures, this study used methods of usability testing and cognitive debriefing in two iterative cycles to establish the acceptability and feasibility of the BAPQ-C with adolescents who experience ongoing pain.

### Participants

A total of 14 adolescents were recruited to this study. Sample size was informed by previous pediatric health assessment tool feasibility studies which have recruited samples of 12–14 participants to complete a newly developed measure [19, 21]. Adolescents were recruited from a specialized UK national pain management treatment center whilst undertaking a 3 week residential pain management programme. Study inclusion criteria required eligible adolescents to be aged 11–18 years, to be attending a current residential pain management programme at the above hospital and have experienced pain for a duration of at least 3 months. Recruitment took place across four consecutive pain management programmes. Over the course of the study, 24 adolescents were approached to take part in the study, with a total of 10 adolescents not providing consent to participate in the study. Reasons for non-participation included leaving the programme prior to completion, insufficient and an internet connection issue which prevented completion of the online measure. Consequently, a total of 14 adolescents (13 females, 1 male) aged between 12 years 7 months and 16 years 6 months (M = 15.1, SD = 1.3) participated in the study. Participant age at symptom onset ranged from birth to 13 years 11 months (M = 7.8, SD = 4.9) and the majority of participants (*n* = 10; 71.4%) reported experiencing pain in 2 or more sites of the body in the last week (see Table 1), with pain most typically reported in the legs (*n* = 12; 85.7%) and head (*n* = 8; 57.1%).

**Table 1:** Description of participants according to diagnosis

Number of participants (% of sample)	Diagnosis	Mean age in years ^a^ (SEM)	Mean number of primary pain site(s)^b a^ (SEM)	Symptom duration in years ^a^ (SEM)	Overall pain level (in last week)^c a^ (SEM)
3 (21.4%)	Pain associated with hypermobility^a^	14.2 (1.2)	2.7 (0.3)	12.7 (0.2)	7.0 (0.0)
6 (42.9%)	Diffuse / localised idiopathic pain	14.9 (0.6)	1.8 (0.5)	3.3 (2.3)	7.7 (0.7)
4 (28.6%)	Complex regional pain syndrome	16.0 (0.2)	2.8 (0.5)	6.1 (1.7)	7.8 (0.6)
1 (7.1%)	Back pain	15.6 (0.0)	2 (0.0)	5.6 (0.0)	8.0 (0.0)

^a^ Standard error of the mean reported in brackets

^b^ Based on score of 5 or above on scale: 1 = no pain to 10 = worst possible pain

^c^ Based on scale: 1 = no pain to 10 = worst possible pain

^d^ Hypermobility is associated with an unusually extended range of movement of joints and pain

## Materials and measures

### Demographic information

Demographic information pertaining to participant sample characteristics was collected via an author designed demographic questionnaire which was completed online prior to completing the BAPQ. Participant age was calculated through participant provided date of birth data, whilst gender was collected through participants completing a tick box to indicate gender (male/female/other). Information about specific pain locations was collected through participants completing a pain body map. Specifically, participants were requested to indicate whether they experienced pain in specific body parts (e.g. front left arm, back of head, front of chest, back right leg). Adolescents were also asked to rate the current pain intensity in affected body parts by completion of a 0–10 numerical rating scale, with 0 representing no pain and 10 representing worst pain possible. Pain body maps have been shown to be effective for use with children aged 8 years and above [30]. The number of pain sites was calculated by summing the number of affected bodily areas where participants provided a score of 1 or greater in terms of pain intensity. To assess overall pain intensity in the past week participants were asked to complete a 0–10 numerical rating scale to indicate their estimation of overall pain within the previous 7 days, ranging from no pain (0) to the worst pain possible [10]. Such 11 point numerical rating scales have been shown to be effective ways to assess pain in children and adolescents [31]. Symptom duration was calculated by requesting participants to record their age in years and months (e.g. 8 years, 7 months) at pain onset. Consequently, symptom duration was calculated by subtracting total age in months at pain onset from total age in months at study completion. Finally, information about diagnoses was collected from clinicians at the time of invitation to participate in the study.

### Bath Adolescent Pain Questionnaire – Computerized (BAPQ-C)

The BAPQ-C is a computerized version of the original paper based Bath Adolescent Pain Questionnaire (BAPQ) [15]. The BAPQ is a multidimensional assessment tool designed specifically for use with adolescents who live with chronic pain. The BAPQ comprises 61 items across seven different domains of functioning affected by pain: physical functioning, social functioning, general anxiety, pain specific anxiety, depression, family functioning and development. The BAPQ is psychometrically robust, with Convergent validity for each subscale and temporal reliability of the measure have also been demonstrated to be high [15]. Cronbach’s alphas for individual BAPQ-C subscales were investigated in this particular study sample and ranged from 0.77–0.91. Specifically, values comprised (1) social functioning (0.86), (2) physical functioning (0.82), (3) depression (0.77), (4) generalised anxiety (0.84), (5) pain-specific anxiety (0.91), (6) family functioning (0.76) and (7) development (0.77).

The sole difference between the previously published BAPQ and the BAPQ-C concerns the online nature of completion of the measure. The BAPQ-C is a web-based questionnaire constructed using Hypertext Markup Language 5 (HTML5). It has a PHP/MySQL backend for data storage and manipulation. As we had a wide range of potential end users and devices, the user interface was designed ‘mobile first’, using a responsive design framework, with optimisations for touch-screen based devices such as tablets and smartphones. The main user interface is a digital representation of the paper-based BAPQ questionnaire, a mix of Likert scales, check lists and free text entry fields. The system is presented as a single web page, with data verification and validation provided by Javascript. All BAPQ items and response options remained the same in the BAPQ-C compared with the original BAPQ. Upon submission of the completed response to the BAPQ-C, the form data was verified for completeness and then stored in a MySQL database. A password protected administration page allowed for the download of database records as an Excel spreadsheet.

### BAPQ-C acceptability questionnaire

This measure comprised three parts and focused on examining how easy participants found the BAPQ-C to complete and requiring participants to compare ease of completion of the BAPQ-C with previous completion of a paper version of the BAPQ (first day of the treatment programme). Individual components of the measure comprised: 1) quantitative Likert-scale questions, 2) qualitative free text questions, and 3) a comparison of ease of completion between the BAPQ-C and paper based BAPQ measures. Specifically, quantitative questions related to how easy the computerized version was to complete (4 response options; ‘very easy (1)’ to ‘very difficult (4)’), how clear the online instructions were (4 response options; ‘very clear (0)’ to ‘very unclear (4)’) and how individuals liked the look of the computerized measure (4 response options: ‘very much (0)’ to ‘not at all (4)’). All responses to these three items were reverse scored and can be found in Table 2. The acceptability measure also included a question to assess perceived time taken to complete the BAPQ-C (three response options: too long (1) to not long enough (3)). Additionally, the acceptability measure comprised three open text questions which asked participants to indicate what they found easy about the computerized version, what they found difficult about completing the measure and any identified problems they experienced when completing the BAPQ-C. The final part of the measure included two response items for each item (BAPQ-C or BAPQ) and asked participants which measure they preferred, which they found the quickest to complete, and which they found the easiest to complete. A final open text question asked participants to provide a free text response to explain why they preferred their selected measure (BAPQ-C or BAPQ).

**Table 2:** Usability characteristics of BAPQ-C

Usability characteristic	Mean score (SD)	Range of scores	Response scale
Ease of completion	3.93 (0.27)	3–4	1 = very difficult to 4 = very easy^a^
Clarity of instructions	3.93 (0.27)	3–4	1 = very unclear to 4 = very clear^b^
Desirability of appearance	3.64 (0.50)	3–4	1 = not at all to 4 = very much^c^

^a^ Reverse scored from survey response scale: 1 = very easy to 4 = very difficult

^b^ Reverse scored from survey response scale: 1 = very clear to 4 = very unclear

^c^ Reverse scored from survey response scale: 1 = very much to 4 = not at all

### Thinking aloud task

A semi-structured thinking aloud task [32] was created to capture participants’ thoughts and feelings about completing the BAPQ-C in real time. The aim of the thinking aloud task was to obtain feedback for the improvement of the BAPQ-C. The thinking aloud task required the researcher to ask questions of the participant as they were completing the BAPQ-C. Questions focused on five key areas which comprised: 1) thoughts regarding computerized presentation and completion, 2) thoughts about individual items and 3) thoughts about domains, 4) thoughts about response choices and 5) overall thoughts regarding completion of the BAPQ-C. Example questions included ‘How easy is it for you to navigate the screens and move onto the next page?’, ‘How did you choose your answer?’ and ‘What do you think about the response choices?’

All participants completed the task, and the responses were audio recorded, and later transcribed by the research team and anonymized.

### Procedure

Participants were informed about the study in the first week of the 3 week residential pain management programme by the clinicians responsible for their treatment and provided with an information sheet. Age appropriate information sheets were provided to all adolescents, with parents of adolescents aged 11–15 years also receiving an information sheet which related to their child’s potential participation in the study. Interested participants were asked to contact the researcher and were given the opportunity to ask questions about the study and their possible participation. Once participants (and parents where relevant) had provided written informed consent and/or assent, an appropriate date and time for participating in the study was mutually arranged between the researcher and the adolescent participant. Parents were required to provide written consent when participants were aged 11–15 years only in accordance with UK healthcare ethical guidance. All participants completed the study session in a quiet room at the hospital.

As each participant had previously completed a paper version of the BAPQ as part of the treatment programme [15] prior to recruitment into the study, each participant only completed the computerized BAPQ-C version of the measure in this study. Throughout the completion of the BAPQ-C, participants were asked questions from the semi-structured Thinking Aloud Task, and the process was audio recorded. Once participants had completed the BAPQ-C and Thinking Aloud Task, they were asked to complete the Acceptability Questionnaire. Before leaving, each participant was debriefed fully, given the opportunity to ask any further questions, and received a £10 Amazon gift voucher as a reimbursement for their time.

### Data analysis

Quantitative data from the BAPQ-C Acceptability Questionnaire was examined using descriptive statistics. Transcribed data from the Thinking Aloud Task and qualitative items on the BAPQ-C Acceptability Questionnaire was analysed using the reflexive principles of thematic analysis advocated by Braun and Clarke [33]. Reflexive thematic analysis is a methodologically flexible approach for identifying patterns in qualitative data to answer a research question. Key elements of Braun and Clarke’s approach to thematic analysis involve conducting processes of data familiarisation, data coding, theme development and revision [34]. Specifically, coding was completed by assigning a code to each text extract and labelling codes according to relevance of the concept and assembling them into potential themes and subtheme categories. Codes were reviewed numerous times. Established relationships between the themes and subthemes were reviewed against the wider data set. Content and labelling of themes and sub-themes was first reviewed independently by F.M.B. and subsequently by A.J. With reference to transparency, a universally approved version of analyses was shared and agreed on by all co-authors, providing credibility checks in terms of analytical interpretation [35]. Additionally, to further explore the issue of ‘quality’ in qualitative research, trustworthiness was established by ensuring that presented quotations were sampled from a range of participants in the study to ensure provision of a wide range of participant accounts. Participants were assigned a pseudonym for the purpose of results reporting. Reported quotes are accompanied by participant pseudonym and participant age (years/months).

## Results

### Quantitative analyses of the BAPQ-C acceptability questionnaire

Participants were asked to rate the usability of the BAPQ-C based on ‘ease of completion’, ‘clarity of instructions’ and ‘desirability of appearance’. Usability characteristics of the BAPQ-C are shown in Table 2. A higher mean score indicates greater ease, clarity and desirability of appearance of the BAPQ-C.

### Qualitative analyses of the thinking aloud task

Two themes were identified in the data: ‘Engagement and technological appeal’ and ‘Accessibility and independence’. These themes are described below and illustrated by a range of quotations derived from participants’ accounts.

### Engagement and technological appeal

In this theme, participants consider the appeal of using technology to complete the computerized BAPQ-C which they would previously have completed on paper. For some participants, the appeal of using technology to complete the questionnaire was initially relatively difficult to articulate; though the computerized BAPQ-C was generally perceived to be more engaging and enjoyable than its paper-based equivalent.

*‘I prefer it. It’s easier to do and it’s very umm, I don’t know really... it’s just very easy to do. And I think it would be quicker than the [paper] questionnaires … ’ (Daisy: 13 years 8 months)*

Other participants were able to provide clearer insights underlying their preference for the computerized BAPQ-C. For the majority of participants, use of a hand-held tablet in everyday life was the norm, and completing the BAPQ-C using this medium was a natural and welcome extension of this. Harriet describes how she and her peer group are attuned to, and engaged by, the use of technology in this context.

*‘Yeah, it’s [BAPQ-C] really good. It’s like really straight forward and it’s like easier to use. I think it will appeal to younger ages because like we’re with technology and we’ve grown up with it.’ (Harriet: 15 years 4 months*)

In particular, Emily highlights a preference for use of technology due to speed of BAPQ-C completion in comparison to completing the paper-based questionnaire (paper version of the BAPQ).

*‘It’s [BAPQ-C] a lot quicker I feel, like I, because of me using my iPad all the time at school I’ve always found it a lot easier... Yeah, so it’s a lot less tedious.’ (Emily: 16 years)*

Participants recognised the importance of completing questionnaires within a healthcare setting and reported that use of technology introduced a level of enjoyment to completion of these necessary measures; alleviating some of the disengagement that they felt when completing paper-based versions of questionnaires.

*‘[Completing BAPQ-C] doesn’t feel like a chore, like you have to do it. Because when everyone gets given the paper, there’s so much paper that everyone like, rushes through it without thinking … they really can’t be bothered to really do it … ’ (Sophie: 16 years 6 months)*

Participants also recognised that the use of technology might present a challenge for some populations, and that a computerized questionnaire might be difficult for individuals who are unfamiliar with this form of media. Amber makes a distinction between her grandmother and herself in terms of their relative abilities to engage with different forms of the questionnaire.

*‘Yeah, I do prefer it, I just think... like my nan, if my nan was to do it I don’t think she... I don’t know, I think she’d find it hard. She doesn’t get on with like, technology and stuff. But I think that if that’s something to get introduced then I think a lot of people might benefit from that rather than the paperwork.’ (Amber: 15 years 9 months)*

### Accessibility and independence

This theme expands more fully on features of the computerized BAPQ-C which increased accessibility when compared to the paper-based BAPQ. Participants cite examples of how practical aspects of the new format supported them in completing the questionnaire more accurately and comfortably, thereby fostering feelings of confidence and independence in their responses to the measure. As reported by Katie and Bethany, discomfort when writing due to their experience of chronic pain was a major consideration for many participants when completing paper-based measures. Use of the BAPQ-C reduced these difficulties to a substantial degree.

*‘Probably better because when you have to tick stuff and if you make a mistake you have to scribble it out but here you can just change it on this. It hurts my hand when I do it on the paper.’ (Katie: 13 years 4 months)*

And

*‘I find it difficult to write as it is, so this is easier.’ (Bethany: 15 years 5 months)*

Participants described other aspects of the BAPQ-C which supported their autonomy in completing the measure. For participants with sight problems in particular, BAPQ-C functions relating to text clarity improved accessibility to the questionnaire and their resultant ability to complete it independently. Sophie explains how use of the ‘zoom’ function allowed her to increase text size in order to read questions more easily.

*‘ … it’s good that you can scroll in and out to make the font bigger and smaller as well.’ (Sophie: 16 years 6 months)*

For Emily, the use of colour allowed her to differentiate more easily between questions and other text, thereby improving the clarity of the questionnaire content for participants.

*‘I struggle with my sight so... I mean it sounds silly but I like the colour. The difference in colours makes it kind-of easy to distinguish what’s a question and what’s a... I mean it’s kind-of obvious but just that bit more I suppose … ’ (Emily: 16 years)*

Similarly, the use of different font styles supported Amber in identifying relevant texts. The added convenience of being able to complete the BAPQ-C without the aid of her glasses facilitated quicker, more accurate understanding and completion of the questionnaire.

*‘ … I can read them without my glasses and that’s like, helpful … I like how it’s in different fonts, like I think that helps like different shape, like the format helps how easy it is to understand.’ (Amber: 15 years 9 months)*

For some participants, functions of the BAPQ-C reduced concerns about their abilities to use vocabulary and spell correctly, and the potentially negative impression that any mistakes might give to others reading their responses. These are important factors given that accuracy of spelling and vocabulary-use in questionnaire completion are not important considerations for clinicians, yet they can be a source of significant worry for adolescents and can present a barrier to questionnaire completion as a result. Participants described how the spell-checking and predictive text functions enabled them to be more confident that such errors would be corrected when completing the BAPQ-C.

*‘I don’t have to worry about spelling. I’m rubbish at spelling!’ (Faith: 15 years 6 months)*

And

*‘... if you’re struggling to find a word, the predictive text is quite helpful because it can try and help you find that word … Yeah, spelling as well. So it’s like … people feel more confident and don’t feel like they’re going to be judged on their spelling if they have difficulties or something.’ (Harriet: 15 years 4 months)*

General features of the BAPQ-C which made the computerized format more accessible than the paper-based BAPQ were also noted by participants. For some, the ability to scroll back and forth through the questionnaire allowed them to navigate the measure easily, without becoming confused about which section they were answering or whether they had missed any questions.

*‘ … it’s easy because it’s just scrolling rather than flipping pages and you’re not sure if it’s double-sided or just one-sided.’ (Milly: 16 years 5 months)*

And

*‘I definitely do prefer that to the paper version. I think, like you do it a lot quicker and [it tells you] if you’ve missed anything out as well, which I think, saves us time than having to move from it and saves you guys [clinicians who request omitted elements of the paper BAPQ be completed] time having to come back to us and stuff.’ (Amber: 15 years 9 months)*

Participants also foresaw the potential for use for the BAPQ-C as an online measure which they could complete from home, thereby increasing their access and the potential for monitoring of their progress and well-being by healthcare professionals.

*‘I wouldn’t have to go out of the house … which would help with lack of mobility. I would be able to do more questionnaires if they were online.’ (Bethany: 15 years 5 months)*

### Quantitative comparison of BAPQ-C and BAPQ

Participants were asked to compare their experience of completing the computerized BAPQ-C with that of completing the paper version of the BAPQ (completed as part of their treatment in the previous week). When comparing the computerized BAPQ-C with the paper-based BAPQ, 13 participants expressed a ‘preference’ for the BAPQ-C (93%) and reported that they found it ‘quicker’ (93%) and ‘easier’ (93%) to complete than the paper BAPQ.

## Discussion

This study is the first to evaluate the feasibility of a computerized version of the BAPQ (BAPQ-C) in a population of adolescents experiencing chronic pain. Through use of cognitive debriefing, participants provided real-time insights into their interactions and experiences when completing the electronic version of the measure. The themes of ‘Engagement and technological appeal’ and ‘Accessibility and independence’ demonstrated a number of factors contributing to participants’ preference for the computerized BAPQ-C compared with the typical paper version of the BAPQ. Study findings highlighted the potential benefits of adopting the BAPQ-C when assessing the impact of chronic pain on adolescents in clinic and home based settings.

Concordant with existing literature which suggests high levels of patient acceptability for health-related measures converted from paper to electronic formats [24–26], particularly amongst younger populations [26], 93% of participants reported that the BAPQ-C was both ‘quicker’ and ‘easier’ to complete than the BAPQ. In fact, only one participant indicated a preference for the paper version of the measure. Whilst this participant reported their preference for the paper-based BAPQ, they also noted their unfamiliarity with the operating system of the mobile device on which the BAPQ-C was being tested. Within a clinical and research setting, it is important to recognise that some individuals may require extra time to adjust to the technology on which the BAPQ-C is presented. More broadly, participants’ preference for the BAPQ-C was driven by generalized considerations regarding their use of and affinity with technology, and more specific functional aspects of the computerized version of the measure that contributed in reducing the burden of completion. Such findings are congruent with the ‘bring your own device’ literature which suggests that self-report health measures can be adapted effectively to electronic formats for online or app-based access via individuals’ existing mobile devices [25, 36].

It is unsurprising that the majority of participants viewed the BAPQ-C favourably considering the ‘digital native’ age-group from which the population was drawn. Recent evidence from a UK-based study of electronic media use in children suggests that 83% of 12–15 year olds own their own smartphone and/or tablet and use these technologies regularly to access a variety of media resources [29]. As technology is normalised in everyday use amongst this population [37], participants viewed the BAPQ-C as more engaging and enjoyable than the paper-based measure. Such factors are likely to be of even greater importance for adolescents experiencing chronic pain. Many of our participants were already using a hand-held tablet as a basic form of assistive technology supporting their day-to-day schooling, and were likely to rely on this technology to facilitate or maintain supportive peer relationships where chronic pain limited their opportunities to socialise in person [38].

Participants noted specific features of the BAPQ-C which supported them in completing the measure. For some, the computerized format of the BAPQ-C allowed them to avoid using a pen to complete tick boxes or correct errors during completion of the assessment; activities which formerly caused them pain, took longer to accomplish, and often required the assistance of others when completing the paper BAPQ. For other participants, the differentiated fonts colours and sizes within the BAPQ-C enhanced readability and understanding, whilst use of the zoom function allowed adjustment of text to accommodate eyesight and reading difficulties. It is notable that these relatively simple functional improvements observed during BAPQ-C completion where not merely seen as increasing the accessibility and acceptability of the measure. They were also experienced in terms of fostering participants’ autonomy and independence; factors which are often restricted in adolescents who experience persistent pain [5]. By providing a format through which adolescents could complete the BAPQ more independently, the BAPQ-C enhanced the potential for participants’ privacy during assessment and reduced the burden of completion as a result. Whether increased privacy might lead to more accurate self-reporting of adolescents’ pain experiences from their own perspective, has yet to be tested. It is possible that the improved accessibility afforded by the BAPQ-C might enable adolescents to provide a more accurate and effective description of the impact of chronic pain on their lives [9, 10] compared with the standard paper version of the BAPQ.

When comparing the computerized BAPQ-C with the paper based BAPQ, participants’ observations of BAPQ-C revealed underlying concerns which they had previously experienced during completion of the paper version. Specifically, some participants were apprehensive about their ability to spell words accurately and use vocabulary correctly, which limited the depth of their responses when completing the paper based BAPQ. These concerns are rarely considered by clinicians or researchers when presenting adolescents with health assessments for self-completion, and have the potential to reduce the scope of chronic pain assessments when using a paper format. For our participants, the spell-checking and predictive text functions of the BAPQ-C increased their freedom in articulating their experiences more confidently when completing the assessment. Supporting confidence and self-efficacy in articulating chronic pain experiences is particularly important for older adolescents as they become more active in decision-making and management of their condition, and during transition from pediatric to adult pain management services [39].

Given the important role that accurate and regular pain assessment plays in the management of adolescent chronic pain [9], the improved accessibility and acceptability of the BAPQ-C amongst participants suggests that the computerized version of the measure would be well-received by adolescents during assessment of chronic pain in clinical settings. Many of the advantages of BAPQ-C completion highlighted from the adolescents’ perspective would equally apply to the clinical and research context. Concordant with existing literature [24, 25], the increased speed and ease of BAPQ-C completion would save time and reduce demands upon staff in clinical and research settings. More comprehensive assessments of adolescent chronic pain would also be possible because the BAPQ-C ‘reminds’ individuals to complete omitted questions, thereby reducing the likelihood of missing data. These improvements, alongside reductions in administrative burden and secondary data entry errors [24–26] have the potential to positively impact clinical assessment and monitoring of chronic pain, and research evaluating the impact of pain management interventions.

## Limitations and future directions

Although this study was successful in evaluating the feasibility of a new computerized version of the BAPQ (BAPQ-C) in a population of adolescents experiencing chronic pain, we acknowledge the study’s limitations. First, whilst sample size was informed by, and compared favourably with, previous pediatric health assessment tool feasibility studies [19, 21], sample size was relatively small and restricted to a group of adolescents attending a 3 week residential pain management programme in a specialized pain treatment center. Further research is necessary to assess the feasibility of the BAPQ-C in less structured contexts such as ongoing pain management monitoring in clinical or home-based settings. Second, whilst participants were able to compare their experiences of completing the paper-based BAPQ with the computerized BAPQ-C because they had completed the former measure as part of the pain management programme that they were currently attending, this paper-based BAPQ data was not available for inclusion in the study. Although it would be of interest to compare data derived from the BAPQ and BAPQ-C, literature suggests that such comparisons are unwarranted where only minor alterations between paper-based and electronic versions of a measure are made [24, 27]; as was the case here. Thirdly, participants were predominantly female (*n* = 13) and within a specific age range (12–16 years). It is important to consider this when contemplating the generalizability of the study findings to wider chronic pain populations, particularly as the BAPQ has been validated for use with adolescents aged 11–18 years [15].

Our findings highlight the increased acceptability and reduced burden of the BAPQ-C for adolescents experiencing chronic pain. Future research might usefully consider wider applications of the BAPQ-C in clinical and research settings. The BAPQ-C has potential as a remote monitoring tool, allowing measurement of adolescents’ pain experiences at home, either between clinic visits or longitudinally in the assessment of pain management interventions. In order to achieve this, the BAPQ-C requires further development and testing as an online measure to ensure that the website is accessible and compatible with a range of mobile devices and operating systems, and is made freely available for use in research and clinical contexts. Furthermore, it is important to consider the development of a downloadable app version of the BAPQ-C to facilitate completion of the measure offline where necessary. Within clinical settings, it is also essential to consider the utility and accessibility of BAPQ-C data and the acceptability of the measure to clinicians. Given the time constraints experienced in busy out-patient consultations, there is a need to develop software which summarises individual BAPQ-C subscale scores and compares these across target populations and across time periods for individual patients, in order to inform clinical decision-making in a simple and meaningful manner.

## Conclusions

This study indicates that the BAPQ-C is a feasible tool for the assessment of functioning in populations of adolescents experiencing chronic pain. The BAPQ-C was well-received by participants. They found the computer-based format of the BAPQ-C to be accessible and enjoyable; and were able to complete the measure more quickly, independently and confidently than the paper-based BAPQ. Increased speed, ease and accuracy of completion make the BAPQ-C an ideal tool for use in busy clinical and research environments. Findings highlight the potential benefits of adopting the BAPQ-C when assessing the impact of chronic pain on adolescents in clinic and home based settings.

## Data Availability

Cognitive debriefing and survey data on which the conclusions of the manuscript rely are presented in the main paper. Full survey data and cognitive debriefing transcripts are available from the corresponding author on reasonable request.

## Abbreviations

BAPQ: The Bath Adolescent Pain Questionnaire
BAPQ-C: The Bath Adolescent Pain Questionnaire – Computerized
